# Specialists’ agreement and construct validation of a visual scale for assessing mucosal healing after third molar extraction

**DOI:** 10.1007/s10006-026-01647-0

**Published:** 2026-10-09

**Authors:** Julia Gomes Lucio de Araujo, Beatriz Campos Pereira, Lais Albuquerque Fernandes, Natacha Kalline de Oliveira, Luciana Correa, Maria Cristina Zindel Deboni

**Affiliations:** 1 School of Dentistry, Oral Surgery Department, Universidade de São Paulo, Av Prof. Lineu Prestes 2227 Cidade Universitária, CEP: 05508-000 São Paulo, Brazil; 2 School of Dentistry, Stomatology Department, Universidade de São Paulo, Av Prof. Lineu Prestes 2227 Cidade Universitária - São Paulo, São Paulo, SP CEP : 05508-000 Brazil

**Keywords:** Third molar, Tooth extraction, Wound healing, Mucosa repair scale, digital clinical images

## Abstract

**Objective:**

To present and validate a digital visual scale for evaluating mucosal wound repair after mandibular third molar extraction. The research seeks to establish a standard-based tool to improve communication in clinical practice and research by reducing subjectivity in postoperative assessments.

**Materials and methods:**

A digital image scale using four clinical inflammatory stages (Scores 0 to 3) of recovering oral mucosa of tooth extractions sites was construct. Employing electronic forms (*n* = 300) were sent to dentists to assess agreement with the consensus score, and construct validity in six tooth extraction mucosal wounds examined by specialists or not. Construct validation involved specialists who evaluated the scale’s utility, graphic quality, and clinical significance via a 5-point Likert scale. There was a total of eighty-three responses for the agreement consensus, and sixty-seven for construct validation.

**Results:**

The scale demonstrated a “fair” inter-rater agreement with a Fleiss’ Kappa of 0.3018. The average agreement was 64.3%, with high agreement (> 80%) for advanced repair stages (Score 3) and greater variability for intermediate stages (Scores 1 and 2). Validation results showed high acceptance, with 96.6% of participants agreeing on the tool’s utility for standardization. There was agreement between specialists and general practitioners. The initial scores may require further descriptive refinement to reduce interpretive overlap but under observer’s calibration it can be used across different dental specialties.

**Conclusions:**

The proposed digital scale showed high professional acceptance and potential usefulness for standardizing the assessment of mucosal healing after third molar surgery.

**Clinical relevance:**

By providing a standardized visual reference, this scale facilitates the early detection of healing deviations or postoperative complications and rationalizes clinical documentation in professional practice.

**Supplementary Information:**

The online version contains supplementary material available at https://doi.org/10.1007/s10006-026-01647-0.

## Introduction

The mandibular third molar surgery is the most widely used model for assessment of acute pain as a primary outcome in randomized clinical trials for new drugs and other therapeutic interventions [1, 2]. In some clinical protocols evaluation of postoperative mucosa wound repair status could be an important clinically relevant outcome to be assessed. The focus of investigations is to recognize clinical deviations from the normality of the healing process and the discomfort that delayed healing could cause the patients recovering. Establishing a reliable measurement method is also essential to improve communication among peers; however, standardizing a valid approach remains a significant challenge [3, 4]. In both clinical settings and research, there are different tools to assess postoperative outcomes, including measures that are both objective and subjective. All measurement instruments, whether quantitative and objective or qualitative and subjective, require calibration or validation to minimize bias and personal interpretation.

In periodontal surgery, there is a very frequent use of the Landry scale, which qualitatively expresses the repair of the gingival mucosa [5]. In oral and maxillofacial surgery, there is no valid and reliable scale to measure the level of repair of the mucosa covering wounds after tooth extraction, particularly in third molar removal. The post-extraction socket wound follows the course of an intramembranous bone repair of an alveolar bone tissue recovered by the gingival mucosa. Usually, it takes two to three weeks for the mucosa to cover the dental socket so that the tissue discontinuity is no longer visible on 14th day [6, 7]. Alveolar bones will take more time to heal or properly regenerate. Variations can happen depending on the type of tooth extracted or on the surgical technique used. Considering an impacted third molar surgery, after minor trauma extraction, individualized inflammatory response occurs within 72 h of surgery and pain; edema; and limitation of mouth opening are normal. After this period, with a variability that can reach up to 7–10 days, the wound should progressively present a decrease in those inflammatory clinical signs, when we should remove suture stitches [8].

The absence of a validated scale has important clinical and scientific implications [9].

It limits the ability of practitioners to systematically evaluate postoperative wound status, detect deviations from normal healing, and communicate findings with agreement among peers [10]. Notably in research, it delays comparability across studies and weakens methodological objectivity in randomized controlled trials, where consistent outcome measures are necessary. Furthermore, the use of digital images represents an innovative approach to overcome these limitations. Digital documentation enables standardization of wound assessment, facilitates training of clinicians and students, and allows multicenter analysis in collaborative studies. This supports current trends in healthcare, where digital tools increasingly support clinical decision-making and scientific evaluation [11].

From a clinical perspective, inadequate mucosal repair can be associated with relevant postoperative complications, such as alveolitis, infection, or suture dehiscence, which impact patient recovery and quality of life. Therefore, a scale that enables early and reliable detection of deviations in the healing process has both practical and preventive value. This study aims to present a useful visual scale of digital images for evaluating mucosal wound repair after mandibular third molar extraction, to support standards-based communication in practice and research.

## Materials and methods

The study was conducted between 2020 and 2021 and was based on the application of electronic forms to specialists or non-specialists’ dentists. The protocol was approved by the Institutional Research Ethics Committee (CEP-FOUSP) under number 4.971.867 in accordance with the Declaration of Helsinki. Acceptance to participation was achieved through the accordance of the examiner after reading the informed consent form at the beginning of the electronic forms. All participants provide their consent before collecting responses. The Ethics Approval Declaration and the consent to participate declaration are as supplementary documents.

### Selection and digital clinical images scale preparation

For the establishment of clinical outcomes scale, standardized digital images of six surgical sites at seventh and at 14th days post extraction mucosa wound of inferior third molar. from files of the Oral Surgery Clinic (Dental School – Universidade de São Paulo) with no local (periodontal disease, dental and nosocomial infections, etc.) or associated systemic comorbidities. Three (LAF; NKO; MCZD) experienced dental surgeons (selected five images at 7 days post-surgery to provide a visual spectrum of diverse levels of inflammatory response to tissue repair. In addition, a sixth image captured fourteen days after tooth extraction was used. This image demonstrated a fully repaired mucosa, serving as a visual reference for a complete mucosa healing and normal tissue appearance. The images corresponded to surgical sites where all procedures were conducted by oral and maxillofacial surgery specialists in adherence to rigorous aseptic protocols and technical approach. Briefly, the inferior third molars were assessed using a mucosal flap created by a sulcular incision and distal relief, and all were extracted after sectioning and osteotomy. After copious saline dental socket irrigation silk sutures were applied to mucosa flap repositioning.

Clinical images were organized by signs such as gingival hyperemia, wound secretion, and inflammation level scored as presented in Table 1.

**Table 1:** Clinical characteristics of mucosa different grades of post operatory healing and scores description

Clinical sign	Grades			
	0	1	2	3
Normal mucosa coloration	Yes	No	No	No
Hyperemia restricted to the wound edges	Yes	Yes	No	No
Hyperemia extending beyond the wound edges	No	No	Yes	Yes
Swelling restricted to the wound edges	No	Yes	No	No
Swelling extending beyond the wound edges	No	No	Yes	Yes
Tissue invagination at the wound site	No	No	No	Yes
Necrosis on the wound surface	No	No	No	Yes
Bleeding	No	No	No	Yes
Purulent secretion	No	No	No	Yes

**Fig. 1:**
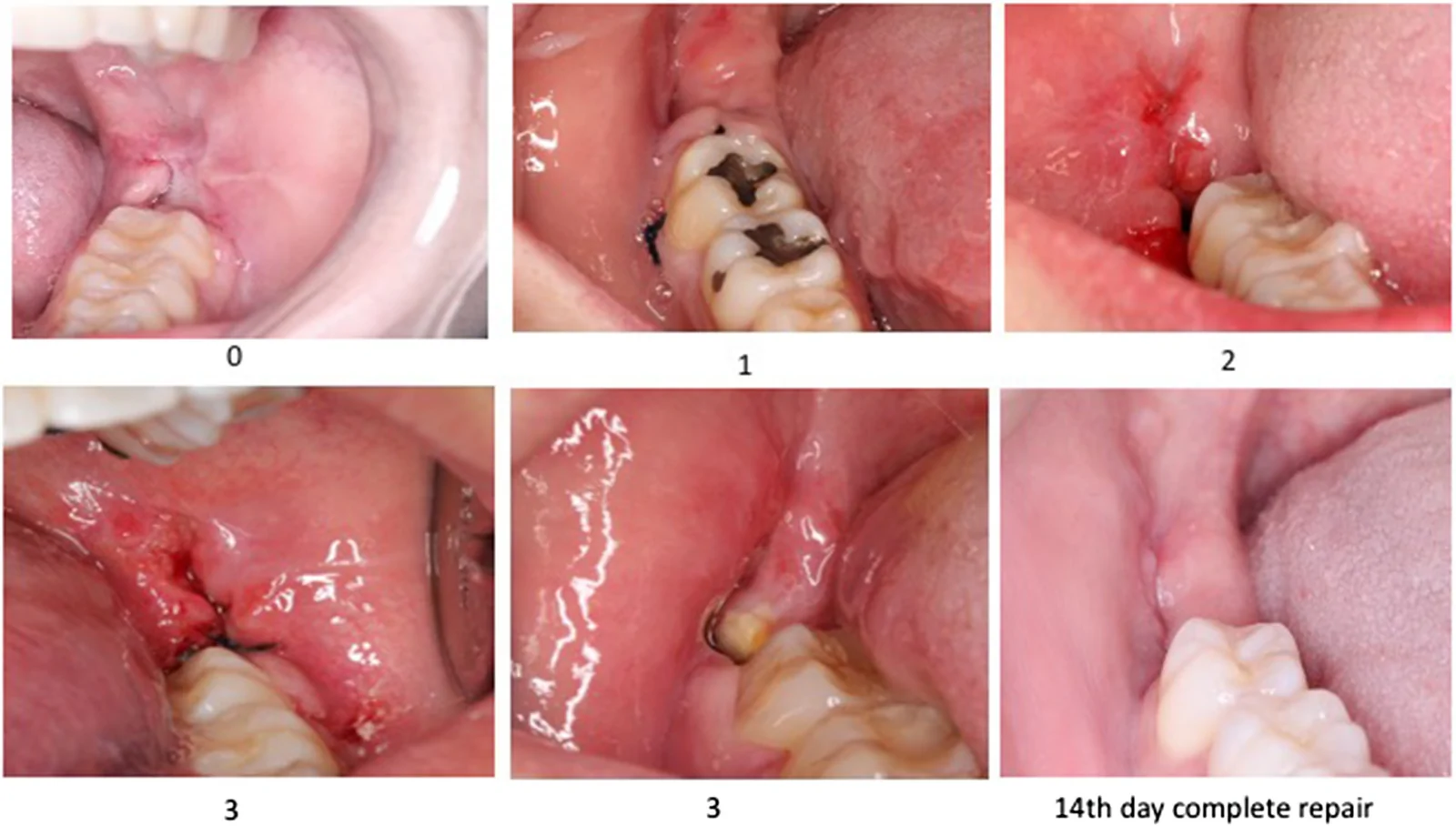
Mucosa wound repair visual scale

The scores and clinical characteristics were adapted from oral mucosal wound repair in periodontal surgery from Aravena et al. [12]. Figure 1 illustrates the mucosa repair scale for third molar extraction.

### Recruitment and eligibility of examiners

Examiners’ electronic addresses were retrieved from a database formed of general dentists and specialists from the records of University Alumni Dental School registers before 2017. The recruitment process ensured a diverse and representative sample of dental professionals. The study consisted of two parallel phases: (1) assessment of the scale’s agreement with the consensus score for mucosal wound repair evaluation, and (2) validation of its construct as a measurement instrument. Both phases were central for assessing the effectiveness and applicability of the scale within clinical and research settings.

In the agreement for consensus phase, 150 dentists with different levels of clinical experience, including both specialists and non-specialists, participated in the study. For the construct validation phase, questionnaires were sent to 150 specialists, including oral and maxillofacial surgeons, implantologists, and periodontists. Specialists from other fields were not included in this phase.

### Evaluation of the agreement by consensus score

**Fig. 2:**
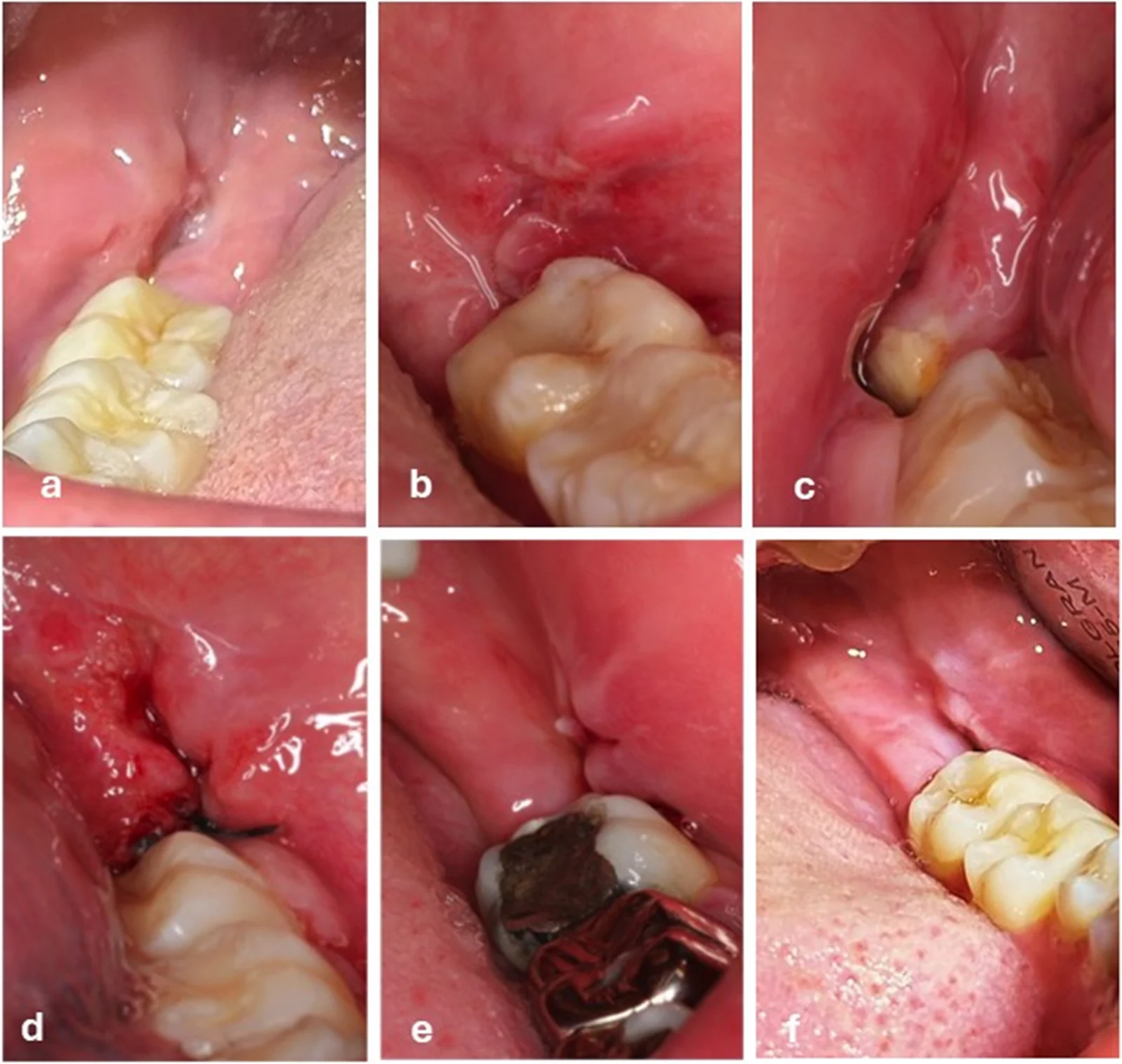
7th and 14th day clinical images after suture removal were used for testing agreement and construct validation of the scale. (**a**) mucosa wound of a right inferior third molar extraction with partially epithelization. (**b**): mucosa wound after a left inferior third molar removal. (**c**) mucosa wound after right inferior third molar removal with a necrotic edge. (**d**) mucosa wound after an inferior left third molar removal with hyperemia and swelling. (**e**) mucosa wound after an inferior left third molar removal partially healed. (**f**) mucosa wound of a left inferior third molar extraction with partially epithelization, slight keratosis and discrete edema

The electronic form assessed the concept, standardization structure, and quality of the scale proposed as a measurement tool for mucosal repair following third molar extraction. The general section provided space to record professional details, such as years since graduation and area of specialization. The proposed scale was introduced through a 1 min and 20 s video, which briefly outlined its application in evaluating the clinical aspects of tissue repair, considering the relevant clinical scoring parameters. Subsequently, the visual scale was shown to participants (Fig. 1). Then they analyzed six further clinical images (Fig. 2) illustrating the mucosa wound on the seventh day postoperative third molar extractions sites. Based on the scale, we requested participants to assign scores of 0, 1, 2, or 3 to indicate the correspondent stage of inflammation and repair of covering mucosa of the tooth extraction sites.

### Scale construct validation assessment

For validation, participants provided professional details (e.g., time since graduation, specialty, experience with third molar extractions). The scale was introduced via a video as described previously. The evaluation used four statements for a five Likers scale assessment: strongly agree, partially agree, neutral, partially disagree and strongly disagree; two direct questions to indicate images, and one open-ended comment query.

1. A scale like this would be useful to standardize the description of mucosal repair of a wound after third molar extraction.
2. The images represent the most common different clinical situations of repair of the alveolar covering mucosa on the seventh day after extraction of the mandibular third molars.
3. The images have graphic/visual quality that allow us to observe the descriptions of the criteria of each score 0, 1, 2 and 3.
4. Mark images that do not have good graphic quality.
5. Mark the images that do not adequately represent the score described by the criteria.
6. The scale can be a reference for the level of inflammation of mucosal wounds for tooth extraction sites in daily practice and in research.
7. Do you want to leave a comment or explain any previous answers? Would you change anything on the scale? Use the space below to enter your text.

### Sample size calculation and statistical analysis

The sample size and stratification were performed to compare and validate interobserver agreement of the scale between different levels of professional experience; the sample was stratified into two independent groups: Specialists in Oral and Maxillofacial Surgery and Traumatology and Non-Specialists. The sample design considered the evaluation of 6 representative clinical images, assuming a type I error of α = 0.05, statistical power of 80% and the detection of a minimum interobserver agreement coefficient (Fleiss’ Kappa) of k ≥ 0.60 (against the null hypothesis). These parameters established the need for at least 50 complete responses per stratum (N_total_=100). Considering variations in the rates of adherence to electronic forms among professional profiles, a response rate of approximately 30% estimated for the group of specialists and 40% for the group of non-specialists. Consequently, the electronic forms were sent to 300 professionals (150 specialists and 150 non-specialists), ensuring the statistical power required for both intragroup analyses and intergroup comparisons.

The agreement with the consensus score and construct validity were evaluated of the proposed oral mucosa repair scale. Inter-rater agreement was assessed using Fleiss’ Kappa coefficient to determine the level of agreement among the examiners across the six clinical images. Agreement with the consensus classification was calculated as the percentage of individual ratings that matched the modal score assigned to each image. The modal score was the internal consensus reference and was not considered an independent gold standard. To examine associations between categorical variables, such as professional specialty and the perceived utility of the scale, we applied the Chi-Square test (x^2^). For, internal correlations between ordinal variables—specifically the relationship between visual quality and clinical representativeness—we used Spearman’s rank correlation coefficient (ρ). Statistical analyses used Python (v3.10). To facilitate data processing and the development of analytical scripts, the Gemini large language model (Google, 2026) was used. The authors verified AI-generated code and statistical outputs to ensure agreement and adherence to the underlying statistical assumptions. The level of significance for all tests was set at α = 0.05. The prompts used to interact with the model are in Supplementary Material.

## Results

**Fig. 3:**
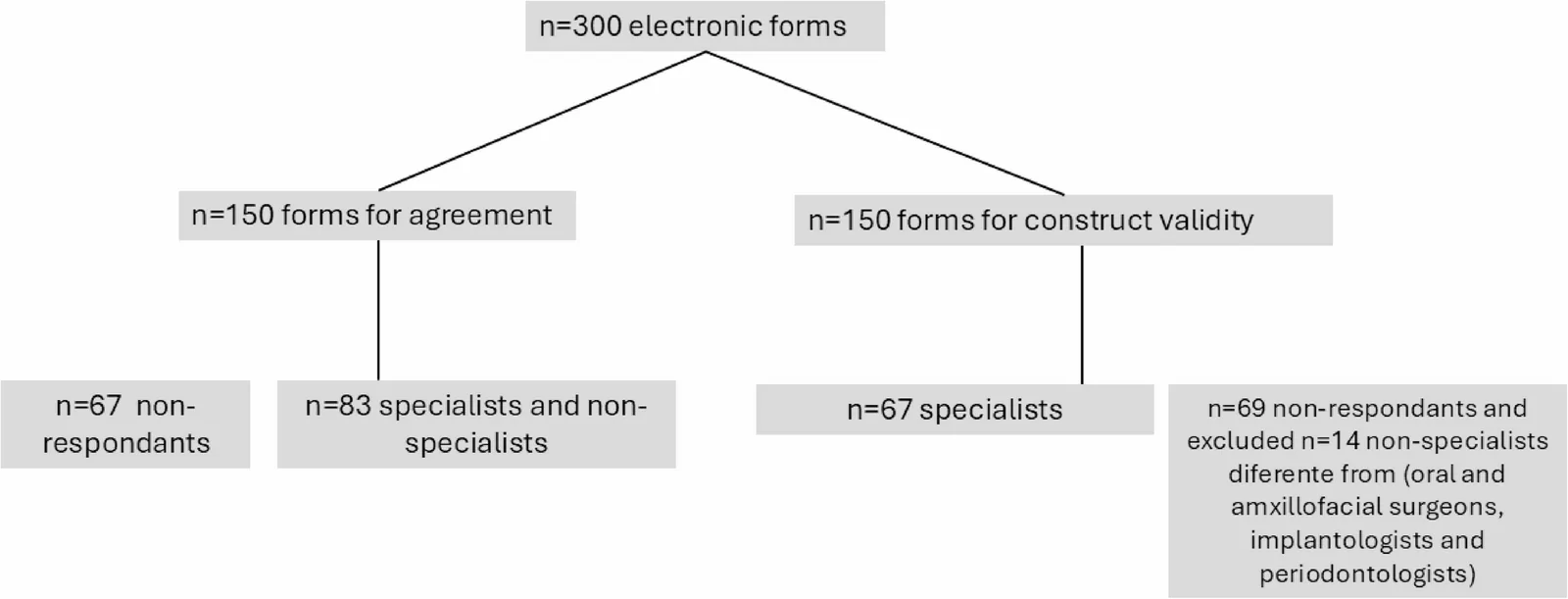
Flowchart of electronic forms distribution and follow-up of respondents

One hundred and fifty examiners answered the three-hundred forms e-mailed. The flowchart on Fig. 3 shows the participants’ distribution.

### Agreement by the consensus score

This analysis evaluates the responses of eighty-three participants in applying the proposed scale to clinical images. The sample included both specialists (31.3%) and non-specialists (68.7%). Among specialists, 25% were oral maxillofacial surgeons, 22% endodontists, 20% periodontists, 20% implantologists, 9% prosthetists, and 4% special care dentists. Kappa Coefficient = 0.3018 according to the Landis and Koch [13] scale, indicates a fair agreement. A correction analysis was measured by calculating the percentage of agreement between individual responses and the Consensus Score (Mode) for each image. The mean ± SD agreement among specialists and non-specialists was 63.5 ± 25.4% and 64.6 ± 21.8% respectively with an overall average of 64.3 ± 22.9%. There was no statistically significant difference in the agreement between specialists and non-specialists (*p* > 0.05). Table 2 illustrates which images reached the highest consensus (higher agreement/correction).

**Table 2:** Consensus agreement for interpretation of clinical images by the scale scores

Image	Consensus score (Mode)	Hits (*n*)	Agreement (%)	Most common error (Score)	Error frequency (%)
c	3	69	83.1%	2	15.7%
d	3	71	85.5%	2	14.5%
f	3	68	81.9%	2	14.5%
a	1	43	51.8%	2	21.7%
b	2	38	45.8%	1	30.1%
e	2	31	37.3%	0	36.1%

Correlations between image ratings were low (< 0.35), which is expected in a validation study where each image serves as an independent test of a specific score. The highest correlation was observed between images 2 and 3 (ρ = 0.35), suggesting a slight tendency for examiners who gave higher scores to one repeat the higher score to the other.

Images representing score 3 (advanced repair) showed elevated levels of correction (> 80%). Conversely, images 5 and 2 showed the lowest agreement, indicating that intermediate repair stages (Scores 1 and 2) are more prone to interpretive variability.

### Scale construct validation

Sixty-seven specialists participated in construct validation. 61.2% were oral and maxillofacial surgeons, 37.3% were implantologists, and 15% were periodontists. It is noteworthy that the participants could point out more than one specialization. 68.7% were considered experienced dental surgeons (Table 3). The scale demonstrated elevated levels of acceptance across all evaluated domains. Most participants “Strongly Agreed” with the proposed criteria, indicating robust face validity.

**Table 3:** Response frequency distribution for construct validation questionary

	Strongly agree	Partially agree	Neutral	Partially disagree	Strongly disagree
Utility for standardization	71.6%	26.9%	1.5%	0.0%	0.0%
Clinical representativeness	80.6%	19.4%	0.0%	0.0%	0.0%
Graphical/visual quality	92.5%	6.0%	1.5%	0.5%	0.0%
Reference for practice/research	70.1%	29.9%	0.0%	0.0%	0.0%

There were minor technical observations. Graphical quality of Image d (Score 3) and Image b (Score 1) were identified by 12% and 10% of participants, respectively, as having lower visual resolution. Spearman’s correlation showed a moderate to strong strength of association (ρ = +0.59) between Standardization Utility vs. Research Reference and a moderate association between clinical reality vs. visual quality (ρ = +0.43). No statistically significant association was found (*p* > 0.05) for any of the primary variables (Utility: *p* = 0.95; Quality: *p* = 0.49).

The open-ended final query provided critical insights for the final refinement of the instrument. Some experts noted that the morphological transition between Score 0 and Score 1 was difficult to detect or analyze, suggesting a need for more distinct visual cues or descriptive descriptors. Suggestions included adding sub-classifications (e.g., 1a, 1b) to account for variables such as “food debris” or “exposed bone tissue.” Several respondents highlighted the scale’s potential to simplify clinical documentation and “electronic oral health records”.

## Discussion

In clinical research, outcomes are measured by instruments or tools that should be valid and reliable for their intention. Construct validation and reliability analysis ensures that the method used has quality and that its application is less susceptible to misinterpretations and biases. Health measurement instruments require an orderly report of their parametric properties. The literature advises that two types of reliability, these being content and construct validities, should be presented in their publication [12]. The validation of a measurement instrument is a methodological procedure in which its quality should be evaluated. The validity may be related to the context in which it is being used, and its variables may be adapted to the theoretical framework adopted. In addition, it should be submitted to judgment, together with the respective completion tool [14]. The purpose of this study was to verify the agreement by consensus of specialists and to construct validate a visual scale of digital images for the standard evaluation of the clinical characteristics of repair of the covering mucosa in tooth extractions sockets particularly of mandibular third molars after 7 days removal.

The results showed the lack of a significant association between the respondents’ experience in oral surgery and the reliability of the scale, suggesting that the acceptance of the scale does not depend on the professionals’ experience with third molar surgeries, making it a potentially applicable tool in different clinical and research contexts. Based on these results, the scale is a promising tool for standardizing and evaluating postoperative follow-up of mucosa wounds from third molar surgeries – a quite common model in clinical trials in oral surgery. In an investigative situation when a clinical outcome is measured, clearly, a calibration step of the assessors should be performed before the use of this tool considering a faithful intraclass correlation coefficient to guarantee reproducibility. In fact, it would be valuable to conduct additional correlation and reliability analyses, in the future, using more robust calibration methods or different test and post-test time points. However, the video here was provided together with the electronic forms for practical reasons, and the materials sent to an anonymous professional database email list. Participants were subsequently identified based on the training and specialization of information provided after completing the forms.

Any tool for measuring the qualities, degree, or quantity of an outcome should be simple and easy to use. To be valid, it must measure what it is proposed to be measured. In the case of wound repair assessment instruments, a clinical description should be proposed in the various phases of the tissue repair process [14]. There is some methods that has been used to evaluate the repair of the oral mucosa, such as the Healing Index (HI) proposed by Landry et al. [5], the Early Healing Index (EHI) developed by Wachtel et al. [15], in addition to the methods of Hagenaars et al. [16], Tonetti et al. [17], and Huang et al. [18], who proposed a method to evaluate periodontal tissue also through scores, the Wound Healing Index (WHI) [5, 15–19]. However, there is no properly standard in outcome assessments in clinical trials especially in third-molar surgeries when the healing of mucosa over the socket is the clinical target. Here, the results demonstrate that the image visual scale proposed had good confirmation by experts. The participation of specialists, with a predominance of oral and maxillofacial surgeons, strengthens the faithfulness of professionals involved in the management of simple to more complex extractions. These specialists considered the scale useful to standardize the description of mucosal repair, with the majority agreeing on its functionality. This suggests that the scale can fill a gap in clinical practice and clinical research tool, providing a standardized visual reference [14]. The suggestions regarding the inclusion of subclasses 1a and 1b for the first score of the scale could be added cautiously to not include confusion as clinical description of extensions of hyperemia or swelling along wound edges.

The proposed scale here did not consider other signs and symptoms related to the inflammatory repair process. It is important to consider that in clinical trial protocol the outcomes with the greatest clinical relevance should be those considered primarily. Pain, trismus, and edema are common clinical conditions associated with the degree of surgical trauma and are obviously of greater clinical relevance to the patient. When patient-reported outcomes (PROS) are associated or correlated with the clinical scores of the scale of repair, the results from the trial could be more relevant to the intervention being tested.

The strongest correlation suggests that professionals who perceive the scale as an effective tool for standardizing clinical descriptions also view it as a viable benchmark for scientific research. Furthermore, the moderate correlation between visual quality and clinical reality indicates that higher image fidelity directly supports the accurate identification of mucosal repair stages. The absence of a significant difference in associations by specialty indicates that the scale could be accepted and interpreted consistently across different dental specialties. However, this finding should not be interpreted as evidence of high cross-disciplinary reliability, because the overall inter-rater agreement remained fair. In oral and maxillary surgery field, some authors presented a scale of measurement for postoperative complications in third molar surgery [12]. The study point out that subjectivity and the need for more precise measures to study the clinical outcome of post-extraction repair become a central point in the discussion about the establishment of a repair scale as a reference. With growing access to the internet around the world, research using the virtual environment is an easy and agile trend for data collection, preferred by most study agents. In this way, this scale can be an auxiliary resource for the exchange and dissemination of information, enables agility in the research process and expands the quality, quantity, and approach of the outcome assessors [20–22].

The newly proposed scale demonstrated high professional acceptance and preliminary evidence of accepting and construct validity. The perception of utility was homogeneous across various specialties, suggesting potential for interdisciplinary use within dentistry [2]. A strong positive correlation indicates that the graphic quality of the images directly supports the identification of repair criteria [3]. Furthermore, the acceptance of the scale as a reference for clinical practice and research would be independent of the professionals’ clinical experience, reinforcing its simplicity and clarity [4].

Notably, there was no statistically significant difference between specialists and general practitioners in their agreement with the consensus scores. This suggests that professional specialization did not influence rating patterns. The images representing the advanced stage of mucosal repair (score 3) exhibited the highest agreement rates. This indicates that the criteria for identifying a well-healed mucosa with minimal inflammatory signs are strong within the scale and easily recognizable by any examiner, regardless of clinical experience. The visual distinction between mild and moderate inflammation understated by certain observers led to interpretive overlap. The most frequent error was the assignment of score zero (0) indicating that a significant part of evaluators confused the initial or intermediate repair stage with a total absence of inflammatory signs, or vice versa. The error patterns reveal a localized tendency for “neighboring” underestimation or overestimation (e.g., scoring 1 instead of 2). In statistics, this is a common phenomenon in ordinal scales and reinforces that, while the scale is a useful tool, the textual descriptors for initial stages of repair could be strengthened. Incorporating more specific clinical markers (e.g., “presence of fibrin” vs. “intact mucosa”) may further enhance diagnostic precision and reduce inter-examiner subjectivity optimizing the instrument’s precision. Although refinement of descriptors in these initial stages of repair could be useful to contrast scores 1 and 2 the inter examiner’s calibration practice in clinical trials outcome measure is not dispensable. The calibration step would certainly diminish overlapping observations.

The six images and the single site of inferior third molars are limitations of the study. However, we chose to use few images evaluations and to focus on a clinically relevant postoperative period commonly used in clinical practice and trials. The seventh postoperative day is a reference point because it corresponds to the usual timing for suture removal or follow-up assessment after one week. Therefore, we aimed to capture routine clinical features typically observed at this stage. We selected the mandibular third molar region because it is a clinical model widely used in oral and maxillofacial surgery and pharmacology trials, particularly for assessing acute inflammatory outcomes such as pain, trismus, and edema. Seven days after mandibular third molar extraction, it should be possible to observe clinical signs consistent with a reduction in the inflammatory phase and the beginning of the proliferative phase. Obviously after 48 h there are important clinical aspects of mucosa repair that would change; though, it will demand another scale regarding a different period of analysis.

New studies could also use optimized similar scales to feed into machine learning systems. AI tools would evaluate on how well the model performs using a grading metric or expert professionals’ feedback; the model parameters or prompt instructions could be based on those grades. In dentistry, especially in oral surgery, it is central that artificial intelligence could frame as a supportive clinical decision-making tool that might work alongside, rather than replace clinical judgment [23]. It is important to point out that AI-assisted approaches carry both profound opportunity and significant risk. Even though, digital tools could introduce substantial risks and ethical concerns regarding the lack of algorithmic transparency, the potential for embedded biases in data training, and the complex liabilities associated with handling personal health data they show the opportunity to enhance diagnostic procedures by automatically presenting pathological edges and enable more objective assessments of mucosal and other tissues wounds among practitioners [24].

Finally, while there is a statistically significant consensus, the “fair” level of agreement suggests that intermediate stages of repair clinical images may present morphological features that overlap between scores, or that the descriptive criteria for certain scores require further refinement to reduce subjectivity. Interestingly, there was no statistically significant difference in the agreement between specialists and non-specialists. The initial scores may require further descriptive refinement to reduce interpretive overlap and external validation could be benefic to the scale in a great and independent sample across dental specialties.

## Conclusion

The proposed visual scale showed high professional acceptance and has the potential to facilitate standardized assessment of mucosal healing after mandibular third molar surgery. Nevertheless, the fair inter-rater agreement observed, especially in the intermediate stages of healing, indicates that refinement of the initial scoring categories and enhanced examiner calibration are required to improve its reliability for clinical outcome evaluation. Future studies could focus on validating the revised scale in larger and more diverse patient populations to enhance its reliability, reproducibility, and clinical applicability.

## Supplementary Information

Below is the link to the electronic supplementary material.

Supplementary Material 1

## Data Availability

The data are available upon request to the corresponding author.

## References

[1] Kim K, Brar P, Jakubowski J, Kaltman S, Lopez E (2009) The use of corticosteroids and nonsteroidal antiinflammatory medication for the management of pain and inflammation after third molar surgery: a review of the literature. Oral Surg Oral Med Oral Pathol Oral Radiol Endod 107:630–640 10.1016/j.tripleo.2008.11.005 19157919

[2] de Santana-Santos T, de Souza-Santos A, Martins-Filho PR, da Silva LC, de Oliveira E Silva ED, Gomes AC. (2013) Prediction of postoperative facial swelling, pain and trismus following third molar surgery based on preoperative variables. Med Oral Patol Oral Cir Bucal.18(1):e65–70. 10.4317/medoral.18039 PMC3548647 23229245

[3] Zandi M, Amini P, Keshavarz A (2016) Effectiveness of cold therapy in reducing pain, trismus, and oedema after impacted mandibular third molar surgery: a randomized, self-controlled, observer-blind, split-mouth clinical trial. Int J Oral Maxillofac Surg 45:118–123 10.1016/j.ijom.2015.10.021 26597577

[4] Martins-de-Barros AV, Barros AM, Siqueira AK, Lucena EE, Sette de Souza PH, Araújo FA (2021) Is Dexamethasone superior to Ketorolac in reduing pais, swelling and trismus following mandibular third molar removal? A split moth triple-blind randomized clinical trial. Med Oral Patol Oral Cir Bucal 26:e141–e150 10.4317/medoral.24088 PMC7980286 33247572

[5] Landry RG, Turnbull RS, Howley T (1988) E ectiveness of benzydamyne HCl in the treatment of periodontal post-surgical patients. Res Clin Forums 10:105–118

[6] Mettes TD, Ghaeminia H, Nienhuijs ME, van der Sanden WJ, Plasschaert A (2012) Surgical removal versus retention for the management of asymptomatic impacted wisdom teeth. Cochrane Database Syst Rev. 10.1002/14651858.CD003879.pub3 22696337

[7] He WL, Yu FY, Li CJ, Pan J, Zhuang R, Duan PJ (2014) A systematic review and meta-analysis on the efficacy of low-level laser therapy in the management of complication after mandibular third molar surgery. Lasers Med Sci 30(6):1779–1788 10.1007/s10103-014-1634-0 25098769

[8] Rodrigues ÉD, Pereira GS, Vasconcelos BC, Ribeiro RC (2021) Effect of preemptive dexamethasone and etoricoxib on postoperative period following impacted third molar surgery – a randomized clinical trial. Med Oral Patol Oral Cir Bucal 24:e746–e751 10.4317/medoral.23095 PMC6901134 31655834

[9] St-Supery V, Tahiri Y, Sampalis J, Brutus JP, Harris PG, Nikolis A (2011) Wound healing assessment: does the ideal methodology for a research setting exist? Ann Plast Surg 67(2):193–200. 10.1097/SAP.0b013e3181f3e0e8 21587059

[10] Bloemen MC, Boekema BK, Vlig M, van Zuijlen PP, Middelkoop E (2012) Digital image analysis versus clinical assessment of wound epithelialization: a validation study. Burns 38(4):501–505. 10.1016/j.burns.2012.02.003 22381563

[11] Casanova-Lozano L, Reifs-Jiménez D, Martí-Ejarque MdM, Reig-Bolaño R, Grau-Carrión S (2024) Evaluation of Two Digital Wound Area Measurement Methods Using a Non-Randomized, Single-Center, Controlled Clinical Trial. Electronics 13:2390. 10.3390/electronics13122390

[12] Avarena PC, Astudillo P, Miranda H, Manterola C (2018) Reliability and validity of measuring scale for postoperative complications in third molar surgery. BMC Oral Health 18:25 10.1186/s12903-018-0486-6 PMC5822564 29466969

[13] Landis JR, Koch GG (1977) The measurement of observer agreement for categorical data. Biometrics 33(1):159–174 PMID: 843571 843571

[14] Zuidgeest M, Hendriks M, Koopman L, Spreeuwenberg P, Rademakers J (2011) A comparison of a postal survey and mixed-mode survey using a questionnaire on patients' experiences with breast care. J Med Internet Res 13(3):e68 10.2196/jmir.1241 PMC3222165 21946048

[15] Wachtel H, Schenk G, Böhm S, Weng D, Zuhr O, Hürzeler MB (2003) Microsurgical access flap and enamel matrix derivative for the treatment of periodontal intrabony defects: a controlled clinical study. J Clin Periodontol 30:496–504 10.1034/j.1600-051x.2003.00013.x 12795787

[16] Hagenaars S, Louwerse PH, Timmerman MF, Van der Velden U, Van der Weijden GA (2004) Soft-tissue wound healing following periodontal surgery and Emdogain application. J Clin Periodontol 31:850–856 10.1111/j.1600-051X.2004.00571.x 15367188

[17] Tonetti MS, Fourmousis I, Suvan J, Cortellini P, Brägger U, Lang NP et al (2004) Healing, post-operative morbidity and patient perception of outcomes following regenerative therapy of deep intrabony defects. J Clin Periodontol 31:1092–1098 10.1111/j.1600-051X.2004.00615.x 15560811

[18] Huang LH, Neiva RE, Wang HL (2005) Factors affecting the outcomes of coronally advanced flap root coverage procedure. J Periodontol 76:1729–1734 10.1902/jop.2005.76.10.1729 16253095

[19] Marini L, Sahrmann P, Rojas MA, Pompa G, Papi P, Pilloni A (2019) Early Wound Healing Score (EHS): An Intra- and Inter-Examiner Reliability Study. Dent. J 7:86 10.3390/dj7030086 PMC6784738 31480586

[20] Norbeck JS (1985) What constitutes a publishable report of instrument development? Nurs Res 34:380–382 3852252

[21] Fleming CM, Bowden M (2009) Web-based surveys as an alternative to traditional mail methods. J Environ Manage 90(1):284 – 92 10.1016/j.jenvman.2007.09.011 18082316

[22] Sussman C (1998) Assessment of the skin and wound. In: Sussman C, Bates-Jensen BM (eds) Wound care: a collaborative practice manual for physical therapists and nurses. Aspen, Gaithersburg, Md., pp 49–82

[23] D´Albis G, Capodiferro S (2026) AI in oral surgery: promise and prudence. Br Dent J 240:12. 10.1038/s41415-025-9509-2) 41513775

[24] Anisuzzaman DM, Wang C, Rostami B, Gopalakrishnan S, Niezgoda J, Yu Z (2022) Image-Based Artificial Intelligence in Wound Assessment: A Systematic Review. Adv Wound Care (New Rochelle) 11(12):687–709 10.1089/wound.2021.0091 34544270

