# Supplementary material for: Specialists’ agreement and construct validation of a visual scale for assessing mucosal healing after third molar extraction
Source: Oral Maxillofac Surg. 2026 Oct 9;30(1):158. doi: 10.1007/s10006-026-01647-0 (PMC13649847; doi:10.1007/s10006-026-01647-0)
Supplement: Supplementary file 1 [file 10006_2026_1647_MOESM1_ESM.docx]

Prompts used for the analyses to be inserted as supplementary material

### **Statistical Analysis Protocols and Methodology**

The following computational and statistical procedures were implemented to analyze the validation and reliability datasets:

#### **1. Data Pre-processing and Categorization**

The initial phase involved data cleaning and the harmonization of variables. Professional specialties were grouped into broader categories (e.g., Oral and Maxillofacial Surgeons, Implantodontists, and Others) to ensure statistical power for association tests. Likert-scale responses regarding utility and clinical reality were mapped to a numerical scale (1 to 4) to facilitate ordinal correlation analysis.

#### **2. Descriptive and Frequency Analysis**

A descriptive study was conducted to determine the distribution of responses for each validation criterion. Frequencies and percentages were calculated to assess the level of professional agreement regarding the scale’s utility, graphic quality, and potential as a clinical reference.

#### **3. Reliability Analysis (Inter-rater Agreement)**

To evaluate the consistency among the 83 independent examiners, **Fleiss' Kappa (k)** was employed. This test was selected as the appropriate measure for multi-rater agreement when using categorical data (the four scores of the scale) across multiple subjects (the clinical images). The resulting coefficient was interpreted based on the Landis and Koch benchmarks.

#### **4. Agreement and Correction Analysis**

The "Correction Analysis" was performed by establishing the **Modal Consensus** (the most frequent score assigned by the total cohort) as the *de facto* gold standard for each image. Individual participant responses were compared against this consensus to calculate:

- **Overall Agreement Rate:** The percentage of correct identifications per image.
- **Error Distribution Analysis:** Identification of the "Most Common Error" to detect systematic misinterpretations between adjacent scores (e.g., confusion between Score 1 and Score 2).

#### **5. Inferential Statistics and Association Testing**

- **Chi-Square Test (X^2^):** Used to determine if there were statistically significant associations between the participants' specialty (or level of expertise) and their perception of the scale. This test aimed to confirm the instrument's cross-disciplinary validity.
- **Spearman’s Rank Correlation (r):** Applied to measure the strength and direction of the relationship between ordinal variables, such as the correlation between the perceived visual quality of the images and their clinical representativeness.

#### **6. Computational Tools**

All analyses were performed using **Python (v3.10)** with the following libraries:

- Pandas and NumPy for data arrangement
- SciPy and Statsmodels for inferential testing and Fleiss' Kappa calculation.
- Matplotlib for the generation of frequency and distribution charts.

The level of statistical significance was set at **p < 0.05** for all hypothesis tests.
